# BUILDING INTERDISCIPLINARY COLLABORATIONS ACROSS THE AGING CONTINUUM: ROLE OF CENTERS ON AGING

**DOI:** 10.1093/geroni/igad104.1239

**Published:** 2023-12-21

**Authors:** Tetyana Shippee

## Abstract

Interdisciplinary collaborations and team science are essential in advancing research across the aging continuum. This involves bringing together experts from different disciplines to work together to address complex topics related to aging. Yet, despite the value of building interdisciplinary collaborations, multiple barriers often exist, that include finding the time to connect, developing shared goals by faculty dispersed across different departments, and developing a commitment to learning from each other and having open communication. This symposium will review multi-pronged strategies to build interdisciplinary collaboration in aging as part of the Center for Healthy Aging and Innovation at the University of Minnesota. In particular, we will discuss the formation of Special Interest groups (SIGs), which have become one key way to encourage interdisciplinary collaborations. SIGs are member driven and are meant to encourage collaboration via joint publications, presentations and grant applications as well as other shared initiatives agreed upon by the group (e.g., resource identifications, book club). Each special group has its own sense of community and draws on members from different disciplines. The symposium will include presentations from four of the seven SIG chairs, focused on the Financial Decision Making SIG, Alzheimer’s Disease and Related Dementias SIG, Transportation SIG, and Aging and Chronic Disease Management SIG. Importantly, the symposium includes perspectives from faculty at different career ranks as well as PhD students.

